# γδ T Cells for Leukemia Immunotherapy: New and Expanding Trends

**DOI:** 10.3389/fimmu.2021.729085

**Published:** 2021-09-22

**Authors:** Mateus de Souza Barros, Nilberto Dias de Araújo, Fábio Magalhães-Gama, Thaís Lohana Pereira Ribeiro, Fabíola Silva Alves Hanna, Andréa Monteiro Tarragô, Adriana Malheiro, Allyson Guimarães Costa

**Affiliations:** ^1^Diretoria de Ensino e Pesquisa, Fundação Hospitalar de Hematologia e Hemoterapia do Amazonas (HEMOAM), Manaus, Brazil; ^2^Programa de Pós-Graduação em Imunologia Básica e Aplicada, Instituto de Ciências Biológicas, Universidade Federal do Amazonas (UFAM), Manaus, Brazil; ^3^Programa de Pós-Graduação em Ciências da Saúde, Instituto René Rachou - Fundação Oswaldo Cruz (FIOCRUZ) Minas, Belo Horizonte, Brazil; ^4^Programa de Pós-Graduação em Ciências Aplicadas à Hematologia, Universidade do Estado do Amazonas (UEA), Manaus, Brazil; ^5^Programa de Pós-Graduação em Medicina Tropical, UEA, Manaus, Brazil; ^6^Instituto de Pesquisa Clínica Carlos Borborema, Fundação de Medicina Tropical Doutor Heitor Vieira Dourado (FMT-HVD), Manaus, Brazil; ^7^Escola de Enfermagem de Manaus, UFAM, Manaus, Brazil

**Keywords:** gamma-delta T cells, leukemic microenvironment, off-the-shelf cell therapy, clinical trials, cell transplantation

## Abstract

Recently, many discoveries have elucidated the cellular and molecular diversity in the leukemic microenvironment and improved our knowledge regarding their complex nature. This has allowed the development of new therapeutic strategies against leukemia. Advances in biotechnology and the current understanding of T cell-engineering have led to new approaches in this fight, thus improving cell-mediated immune response against cancer. However, most of the investigations focus only on conventional cytotoxic cells, while ignoring the potential of unconventional T cells that until now have been little studied. γδ T cells are a unique lymphocyte subpopulation that has an extensive repertoire of tumor sensing and may have new immunotherapeutic applications in a wide range of tumors. The ability to respond regardless of human leukocyte antigen (HLA) expression, the secretion of antitumor mediators and high functional plasticity are hallmarks of γδ T cells, and are ones that make them a promising alternative in the field of cell therapy. Despite this situation, in particular cases, the leukemic microenvironment can adopt strategies to circumvent the antitumor response of these lymphocytes, causing their exhaustion or polarization to a tumor-promoting phenotype. Intervening in this crosstalk can improve their capabilities and clinical applications and can make them key components in new therapeutic antileukemic approaches. In this review, we highlight several characteristics of γδ T cells and their interactions in leukemia. Furthermore, we explore strategies for maximizing their antitumor functions, aiming to illustrate the findings destined for a better mobilization of γδ T cells against the tumor. Finally, we outline our perspectives on their therapeutic applicability and indicate outstanding issues for future basic and clinical leukemia research, in the hope of contributing to the advancement of studies on γδ T cells in cancer immunotherapy.

## Introduction

The leukemic microenvironment is composed of a complex and distinct network of factors that strongly support the growth and clonal dissemination of leukemic cells (LCs), thus impacting the patient’s clinical outcome (1–4). In this context, whereas conventional T cells (CD4^+^ or CD8^+^) and natural killer cells (NK) have been reported as “cytotoxicity mediators” capable of inducing tumor regression *in vivo* and controlling leukemic proliferation, several reports pointed to the fact that other T cells considered “unconventional” also have a high potential for coordinating the immune system and play complex and promising roles in cancer immunity (5–9). These antitumor responses are generally mediated by individual molecules with high or low diversity, such as the alpha-beta (αβ) or gamma-delta (γδ) T cell receptor (TCR) (10, 11).

In contrast to the αβ TCR, which is highly reactive to polymorphic molecules of the major histocompatibility complex (MHC), γδ TCR-expressing T cells perform their functions through recognition of antigens (Ags) presented by several monomorphic molecules, which in turn, promote a strong, rapid and effective response (12–14). In addition to being evolutionarily conserved, γδ T cells are important effectors, since they link innate and adaptive immune responses (11, 15, 16), and are highlighted as promising targets in cancer immunotherapy, especially for leukemias. These hematological malignancies are highly heterogeneous and are defined based on blast count, maturation stage and flow cytometry immunophenotyping, which allows them to be generally classified in acute lymphoblastic leukemia (ALL), chronic lymphocytic leukemia (CLL), acute myeloid leukemia (AML) and chronic myeloid leukemia (CML) (4, 17–23).

A potential therapy against these malignancies may depend on the mobilization and targeting of effector immune cells capable of producing antitumor factors and effectively killing LCs in different compartments with the absence of toxicity or alloreactivity. In this context, γδ T cells have unique attributes that support the promising development of an off-the-shelf cell therapy, as these lymphocytes provide a lasting and efficient response through mechanisms that include a higher cytotoxicity, functional plasticity, the production of several soluble molecules and responsiveness independent of MHC/HLA expression (24, 25). Although the tumor microenvironment (TME) and the adjacent LCs may develop several strategies to escape from γδ T cell-mediated immune surveillance, *ex vivo* or *in vivo* activation, the expansion and the genetic modification of these lymphocytes may increase their antileukemic reactivity and overcome suppression and resistance established by the TME (1, 26).

There is emerging evidence that γδ T cells exhibit persistent antitumor responses in different compartments in patients with leukemia and preserve healthy tissues; however, the adjacent mechanisms are still poorly understood (27–31). Therefore, γδ T cells are being translated into several clinical and therapeutic strategies targeting these hematological malignancies. Herein, we integrate the current knowledge regarding the diversity of γδ T cells and their associated potential in leukemia immune surveillance. Several approaches to improve their antitumor functions allow effective targeting against LCs and, therefore, will be discussed here. Finally, we emphasize open questions about γδ T cells and their subtypes, and also highlight their therapeutic applicability against leukemia. A better understanding of the functional relevance of γδ T cells in these malignancies has important implications, as we may be close to the unprecedented ascension of T cell-based therapies and their positioning as key-components for improving immunotherapy against cancer.

## Untangling the Riddle of γδ T Cell Diversity

γδ T cells make up a lymphoid lineage that has relevant functions in tissues and blood circulation. Their development is regulated in the thymus, where they undergo maturation in different stages of thymic ontogeny (32–34). In this process, genetic rearrangements define the compromise and differentiation of double-negative thymocytes (CD4^-^ and CD8^-^) for the T cell lineage-expressing γδ TCR (35–38). Subsequently, these cells migrate to peripheral blood (PB) and mucosal tissues, where they play key roles in the host’s immunity as primary effectors in the response against infections and cancer (15, 39, 40), preceding the responses of the αβ T cell lineage (41).

Currently, four major subtypes of human γδ T cells have been documented, which are defined by the TCR δ chain (i.e., Vδ1, Vδ2, Vδ3 and Vδ5) according to the Lefranc & Rabbits’s system nomenclature (42). Vδ1 and Vδ2 subtypes are the most predominant (11, 43–45). Vδ3 cells make up the majority of Vδ1^-^/Vδ2^-^ subtypes and are rarely found in PB, although they are found in large numbers in the liver (46). Vδ5 cells also can be found in PB or tissues, but their functions are not entirely clear (43, 47–50). Here, we will focus on Vδ1, Vγ9Vδ2 and Vδ3 cells that are primarily thought to be involved in antileukemic responses.

Overall, γδ T cells constitute up to 10% of circulating CD3^+^ cells, though predominate among all tissue-resident T cells (51–54). Vδ1 and Vγ9Vδ2 subtypes represent ~10% and 90% of blood γδ T cells, respectively (51, 55, 56). While polyclonal Vδ1 cells are distributed throughout tissues, and exhibit adaptive-like behavior after detection of metabolic Ags and stress-induced molecules, Vδ2 cells predominate in blood and exhibit innate-like behavior after detecting molecules named phosphoantigens (pAgs) and other non-peptide antigens (11, 39, 57–59). A minor subtype of Vδ3 cells makes up ~0.2% of total circulating T cells and recognize CD1d and annexin-A2 (ANX2) (49, 60). In addition, little-known subtypes include Vδ5 cells, which detect the endothelial protein C receptor (EPCR), and other distinct clonal populations such as Vδ4, Vδ6, Vδ7 and Vδ8 (43, 61–63). Nonetheless, the enigma of the combinatorial and functional diversity of γδ TCRs has been partly revealed only for the Vδ1 and Vγ9Vδ2 subtypes **(** **Table 1** **)**.

**Table 1:** Diversity of human γδ T cells.

Subtype	Paired Vγ gene usage	Tissue distribution	Major secreted effector molecules	Major recognition receptors	Activation stimulus or TCR ligand	Ref.
Vδ1	Vγ2, Vγ3, Vγ4, Vγ5, Vγ8, Vγ9, Vγ10, Vγ11	Skin, gut, liver, spleen, lung, PB and BM	IFN-γ, TNF, IL-4, TGF-β and IL-17	TCR, TRAIL, FasL, NKG2D, NCR, FcγRIII and 2B4	Lipid Ags, MIC-A/B, ULBP, NCRL, CD1, MR1 and BTNL	(13, 29, 64–70)
Vδ2	Vγ9	PB, spleen, BM and LN	IFN-γ, TNF and IL-17	TCR, TRAIL, FasL, NKG2D, DNAM-1, TLR, FcγRIII and 2B4	pAgs, BTN, BTNL, N-BPs, MICA/B, ULBP, PVR and Nectin-2	(71–75)
Vδ3	Vγ2, Vγ3, Vγ8	Liver, gut, PB, BM and LN	IFN-γ, TNF, IL-4 and IL-17	TCR, FcγRIII and NKG2D	CD1d and ANX2	(46, 49, 60)
Vδ4	Vγ6	PB	ND	ND	ND	(61–63)
Vδ5	Vγ4	PB	IFN-γ and TNF	TCR	EPCR	(43)
Vδ6	ND	PB	ND	ND	ND	(61–63)
Vδ7	ND	PB	ND	ND	ND	(61–63)
Vδ8	ND	PB	ND	ND	ND	(61–63)

An expanded view of human γδ T cell subtypes allow us to observe that their diversity is principally dictated by the individual variations of γδ TCRs and the diversity of their co-receptors. The TCR repertoire of Vγ9Vδ2 cells is the best known and targets butyrophilin (BTN) proteins, for example, which undergo a spatial and conformational change in the target cell membrane, and activate these lymphocytes in a phosphoantigens (pAgs)-dependent fashion. In contrast, non-Vδ2 TCRs are still poorly studied, although some ligands have been discovered, namely, CD1, MHC class I related protein (MR1) and the endothelial protein C receptor (EPCR), which can be expressed in cancer cells. Additionally, cell activation is not mediated only by γδ TCR binding to their cognate ligand, but optionally requires the engagement of co-receptors, such as DNAX accessory molecule-1 (DNAM-1) and natural cytotoxicity receptors (NCR), which results in the high production of effector molecules.

ANX2, annexin A2; BM, bone marrow; BTNL, butyrophilin-like; FasL, human apoptosis-related factor ligand; FcγRIII, Fc gamma receptor III; LN, lymph node; MICA / B, MHC class I chain-related antigens A and B; N-BPs, aminobiphosphonates; NCRL, NCR ligand; ND, not determined; NKG2D, natural killer group 2 member D; PB, peripheral blood; PVR, polyoma virus receptor; TCR, T cell receptor; TLR, toll-like receptor; TRAIL, tumor necrosis factor-related apoptosis-inducing ligand; ULBP, UL16-binding proteins.

## γδ T Cells and Leukemia: The Leukemic Microenvironment Matters

Basic scientific discoveries regarding leukemia have revealed that LCs adopt numerous mechanisms for evading immune surveillance (1, 76, 77). This cancer cell hallmark involves a heterogeneous group of components i.e., stromal and/or immune cells, specific receptors and soluble molecules that are present in the leukemic microenvironment, and which reprogram the hematopoietic niche and promote the clonal expansion of LCs in the bone marrow (BM). The subsequent tumor overload in this compartment results in the release of LCs into the blood, constituting two important sites of high leukemic clonal proliferation (1, 4, 26, 78). This is because LCs can bypass antitumor responses and, consequently, develop a high potential for making the environment extremely tolerogenic (79–81). For this, they adopt intrinsic and extrinsic strategies that impair the immune response of T cells and NK cells (26, 77, 82). Among these strategies, the negative regulation of HLA expression, high expression of inhibitory ligands for programmed cell death 1 (PD1), cytotoxic T lymphocyte antigen 4 (CTLA4) or lymphocyte activation gene 3 (LAG3) and the production of regulatory factors (i.e., cytokines, chemokines and inhibitory enzymes) are important changes that contribute to the inhibition of antitumor cells and the recruitment of suppressor cells that support the survival of LCs (83–99).

**Figure 1:**
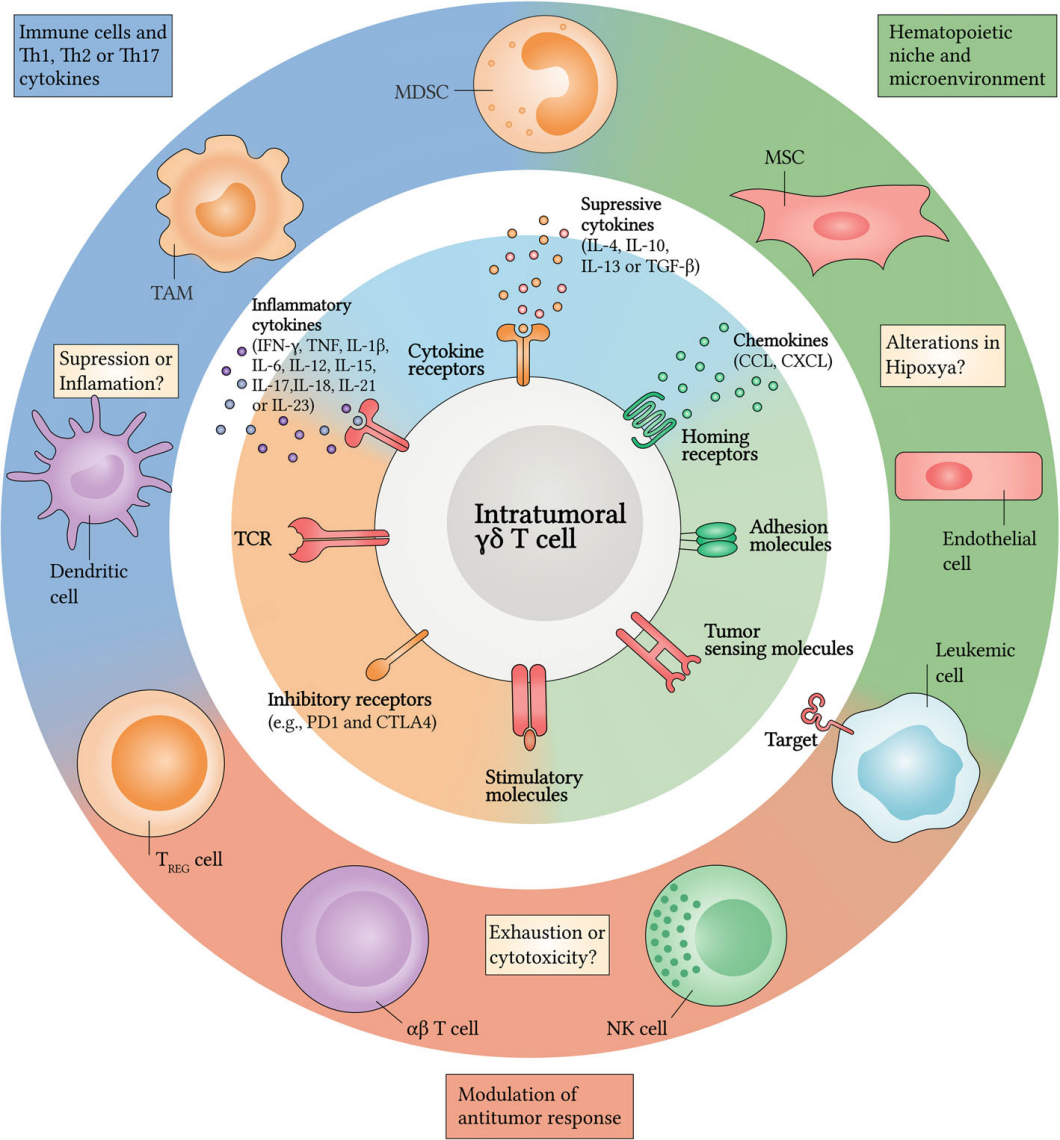
Crosstalk between γδ T cells and the leukemic microenvironment. Upon infiltrating the TME, γδ T cells are exposed to several persistent inflammatory and/or suppressive signals. Pathways implicated in crosstalk with the leukemic microenvironment can be classified into three general categories (center and inner circle): (i) cell-to-cell signals including antigen recognition by γδ T cell receptor (TCR), stimulatory or inhibitory molecules and/or tumor-sensing molecules; (ii) soluble factors such as cytokines and chemokines that will drive changes in expression levels of (iii) homing receptors and adhesion molecules. Several stromal and/or immune cells could be the source of many of these changes (outer circle). Among these, tumor-associated macrophages (TAM), myeloid-derived suppressor cells (MDSC), regulatory T (Treg) cells and dendritic cells (DC) retain their reprogramming potential into the TME by regulating inflammation or suppression through Th1, Th2 and Th17 cytokines. In addition, the hematopoietic niche can regulate hypoxia, responsible for supporting leukemic cells (LC) survival. Mesenchymal stromal cells (MSC) and endothelial cells can also express many factors that attract antitumor cells, such as γδ T cells, αβ T cells and NK cells which can exert cytotoxicity or undergo cell exhaustion after infiltrating the leukemic microenvironment. CCL, CC-chemokine ligand; CTLA4, cytotoxic T lymphocyte antigen 4; CXCL, CXC-chemokine ligand; PD1, programmed cell death protein 1.

These established modifications in the leukemic microenvironment have great capacity for modifying cellular functions and for suppressing antileukemic responses – a consequence of the increase in components, such as regulatory T (Treg) cells, immunosuppressive myeloid cells (IMC), mesenchymal stromal cells (MSC) and inhibitory proteins (e.g., PD1 and CTLA4), which have a high regulatory influence (1, 26). γδ T cells are not exempt within this context, since they are susceptible to the effects of several molecules such as interleukin (IL)-4, IL-6, IL-13, IL-17, IL-23 and transforming growth factor beta (TGF-β) (100–105). These factors can play synergistic or pleiotropic roles, and can induce γδ T cell exhaustion or their polarization into a tumor-promoting phenotype **(** **Figure 1** **)**, thus contributing to malignant progression (24, 106–109).

Although LCs can escape the immune surveillance of αβ T cells and NK cells, they have several molecular targets that can be detected by γδ T cells; however, the crosstalk between these lymphocytes and the leukemic microenvironment is still poorly understood **(** **Figure 1** **)**. Initially, γδ T cell responsiveness does not depend on MHC expression by LCs, whereas conventional αβ T cells require the MHC-Ag axis for activation to occur. The restricted specificity of conventional αβ TCR is also an important factor to be considered, as it is restricted to the detection of peptide antigens. In contrast, γδ TCR can identify stress-induced molecules, pAgs, lipid Ags and many other non-peptide molecules (110). In the context of leukemias, these attributes may offer an unconventional response pathway against these hematological malignancies.

### Mobilization and Recruitment of γδ T Cells Into the TME

The pattern of γδ T cell migration and recruitment has not yet been fully characterized in the context of cancer and, therefore, represents an important question to be investigated. In humans, Vδ1 cells up-regulate the expression of CC-chemokine receptor 2 (CCR2) and CXC-chemokine receptor 3 (CXCR3) and infiltrate the TME. They are also activated by CC-chemokine ligand 12 (CCL2) and CXC-chemokine ligand 10 (CXCL10) and exhibit higher IFN-γ production (111, 112). Furthermore, Vδ1 cells express CXCR1 strongly and CCR5 weakly, whereas their Vγ9Vδ2 counterpart only exhibit strong expression of CCR5 (113). Interestingly, the CCR4/CCR8–CCL17/CCL22 pathway has also been shown to be an additional axis of chemoattractant signaling that recruits Vδ1 cells to the TME (114). It is important to note that Vγ9Vδ2 cells, besides retaining a high expression of CCR5, also express CCR3 and CXCR3, and can trigger antitumor responses in peripheral tissues during metastasis (115, 116).

A more accurate analysis of the profile of homing receptors expressed by γδ T cells would reveal how these cells migrate to the bone marrow microenvironment, for example. It is known that the mobilization of immune cells in this compartment is mediated mainly by the CXCR4-CXCL12 pathway, and it has been shown that CXCR4^+^ γδ T cells (preferably Vδ1 cells) respond to CXCL2 *in vitro*, but their intramedullary homing abilities have not yet been evaluated in the *in vivo* context of leukemia (117–119).

Despite this, many *in vitro* studies have shown that γδ T cells recognize and destroy leukemia blasts, but the complex network of interactions with the tumor environment *in vivo* remains poorly elucidated (120–122). A comparative analysis suggested that Vδ1 TCR-expressing γδ T cells were the most frequent subtype in the BM of pediatric patients with ALL (123). Subsequently, a low circulating γδ T cell frequency was detected in patients with AML before chemotherapy. Patients who regressed to minimal residual disease exhibited higher γδ T cell frequencies, whereas patients with a high leukemic burden exhibited decreased counts (27).

Transcriptomic analyses revealed an abundance of tumor-infiltrating Vγ9Vδ2 cells in cohorts of patients with leukemia (124). This high frequency was positively correlated with the survival of these patients. Although these results are encouraging, the method used to determine the relative proportions of these cells has failed to differentiate them correctly from αβ T cells and NK cells. As a result, this may have contributed to a higher γδ T cell count.

Vδ1 cells have been reported to have increased percentages in patients with CLL (28–31). A high frequency of these cells has been shown to be directly proportional to leukemic progression, that is, patients in more severe states exhibited higher Vδ1 cell counts when compared to healthy patients. This allows these lymphocytes to constitute the major γδ T cell subtype in the PB of these patients, where Vγ9Vδ2 cells generally predominate. This finding was also accompanied by cytotoxic Vδ1 cells with high granzyme (Gzm) B expression (28). Taken together, these data suggest that leukemia affects the γδ T cell frequency and that these cells have some influence during disease regression or progression.

On the other hand, a higher Vγ9Vδ2 cell frequency was associated with a poor prognosis in patients with untreated CLL (125). These lymphocytes showed a dysfunctional phenotype with reduced expression of NKG2D, although the derived LCs showed a high pAgs synthesis. This suggests that Vγ9Vδ2 cells expand in patients with leukemia and may exhibit functional exhaustion, apparently after long-term exposure to pAgs produced by LCs. Based on these reports, it becomes clear that the precise frequency of these cells and their clinical significance during the progression of leukemia is still controversial. In addition, the few studies carried out again suggest that the microenvironment of these malignancies has a strong influence on γδ T cells.

### The Leukemic Cell–γδ T Cell Interactome

**Figure 2:**
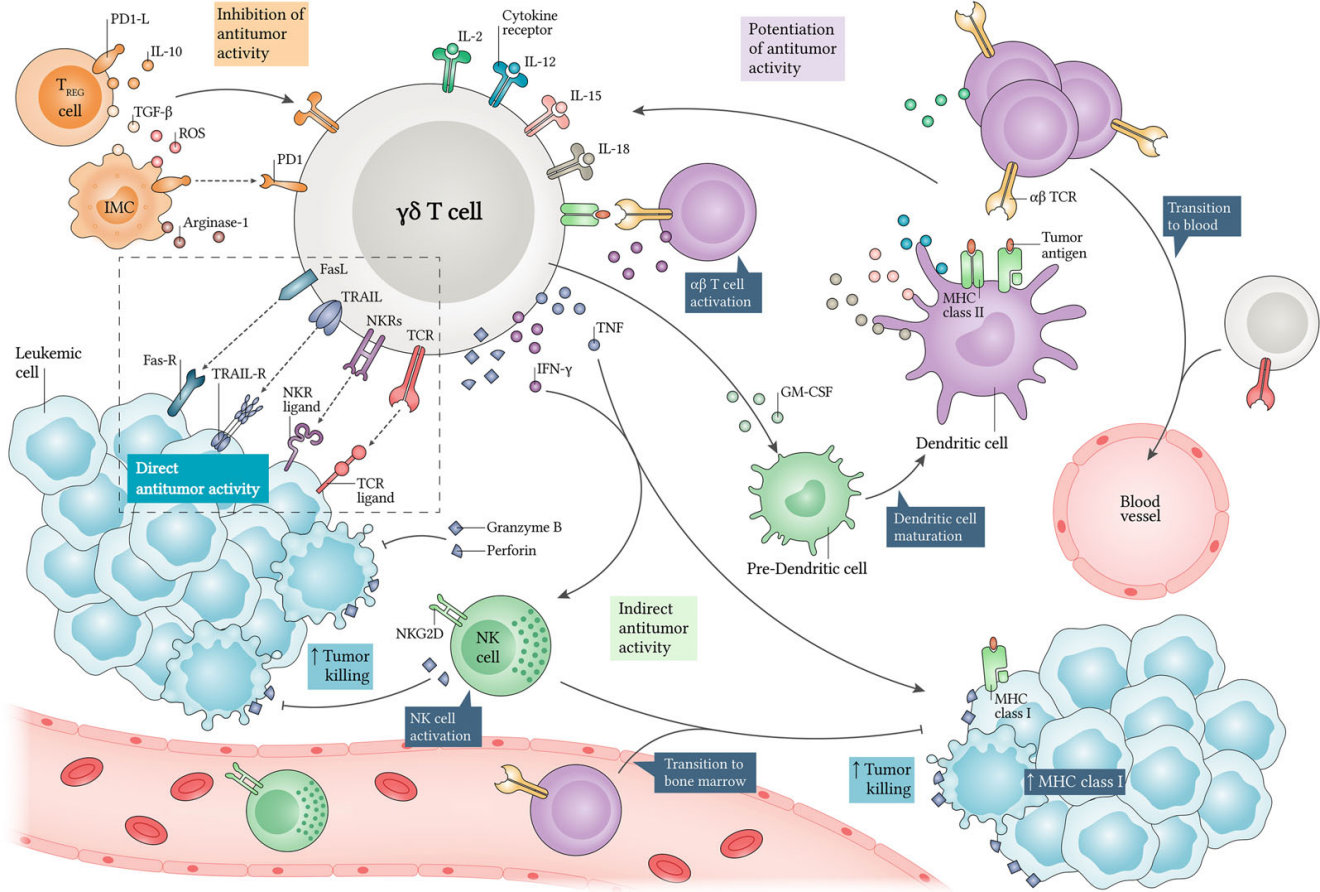
Antileukemic roles of γδ T cells and their regulation. γδ T cells kill leukemic cells (LC) *via* direct and indirect mechanisms. When identifying LCs through γδ TCR and co-receptors such as natural killer cell receptors (NKR), they secrete high levels of perforins and granzymes, mediating direct target killing. Additionally, γδ T cells produce interferon (IFN)-γ and tumor necrosis factor (TNF), which can increase MHC class I expression in LCs, and enhance αβ T cell-mediated cytotoxicity. IFN-γ release also allows NK cell activation, which can enhance tumor killing *via* NKG2D. Alternatively, γδ T cell-derived granulocyte-macrophage colony-stimulating factor (GM-CSF) can induce dendritic cell (DC) maturation, which in turn potentiates antitumor responses *via* interleukin (IL)-2, IL-12, IL-15 and IL-18. Thus, αβ or γδ T cells and NK cells can be recruited for exerting cytotoxicity in many compartments. Moreover, γδ T cells display APC functions and support αβ T cell and NK cell polarization towards an antitumor phenotype. In contrast, their cytotoxicity can be decreased by regulatory T (Treg) cells and immunosuppressive myeloid cells (IMC), since they produce several inhibitory factors such as IL-10, transforming growth factor β (TGF-β), reactive oxygen species (ROS) and Arginase-1. Finally, PD1-PD1L axis expression can regulate γδ T cell antitumor activities. APC, antigen-presenting cell; FasL, Fas ligand; Fas-R, Fas receptor; PD1, programmed cell death protein 1; PD1-L, PD1 ligand; TRAIL, tumor necrosis factor-related apoptosis-inducing ligand; TRAIL-R, TRAIL receptor.

The sensing of LCs and γδ T cell activation are attributed to antigen recognition by γδ TCR and/or NK cell receptors (NKR), which include the natural killer group 2 member D (NKG2D) receptor, for example **(** **Figure 2** **)**. Several reports have shown that LCs express several NKG2D ligands, which include stress-induced molecules, such as MHC class I chain-related protein A (MIC-A), MHC class I chain-related protein B (MIC-B) and UL16-binding proteins (ULBP) (71, 126, 127), while the lack of expression of these ligands is high related to immune evasion of LCs (128, 129). Besides this, some γδ T cell subtypes have a well-documented role in promoting NKG2D-mediated antileukemic responses.

Vδ1 cells recognize and destroy ULBP3^+^ MIC-A^+^ LCs and produce higher concentrations of interferon (IFN)-γ and tumor necrosis factor (TNF) in response to the tumor (29). In parallel, Vδ2 cells detect high regulated ULBP1 in LCs and this is indicative of tumor susceptibility to the cytotoxicity of these lymphocytes (130–132). It has also been established that Vδ1 and Vδ2 cells can destroy ULBP2^+^ LCs (133). Although an almost undetectable ULBP4 expression has been reported in leukemias (129, 134), remarkably, it has been shown that Vδ2 cells detect this molecule in LCs and respond with potent cytotoxicity (135). Therefore, the NKG2D receptor plays a key-role in γδ T cell-mediated immune surveillance in leukemia.

In addition to the expression of stress-induced molecules, an uncontrolled synthesis of metabolic molecules by cancer cells has emerged as a target that can be detected exclusively by reactive γδ T cells, such as the pAgs identified by Vγ9Vδ2 TCR. The pAgs detection mechanism involves butyrophilin (BTN) molecules, which are proteins related to the B7 family of co-stimulatory molecules. BTNs are essential prerequisites in γδ T cell activation, as they perform the intracellular capture of pAgs, undergo spatial and conformational changes in the membrane surface of target cells and consequently bind to the Vγ9 and Vδ2 TCR chains, sending strong stimulatory signals (72, 73). Thus, BTN3A2 has been shown to mediate the recognition of leukemic blasts even though it does not have the B30.2 intracellular domain, important in the internal pAgs uptake (136, 137). This suggests that BTN3A2 can recruit other isoforms, such as BTN3A1 or BTN3A3, and send activation signals through their intracellular domains (138). It is important to highlight that the presentation of pAgs by BTN proteins is highly regulated in LCs, whereas in normal cells the opposite occurs (139).

γδ T cells can also identify specific Ags in the context of monomorphic MHC class I molecules, such as the CD1 protein family (64). These proteins can mediate endogenous or exogenous lipid Ags recognition by γδ TCR and can be detected without loading with lipid Ags (140–142). Two major subtypes of CD1-reactive γδ T cells have been identified, namely Vδ1 and Vδ3 cells (60, 143). It is well established that these molecules are expressed in LCs and exhibit different expression patterns that are related to the leukemia subtype (144). In this context, γδ T cells may play important roles against LCs through the recognition of CD1 proteins and their isoforms.

In fact, CD1 proteins have established themselves as mediators of γδ T cell antitumor responses (145). It is important to note that the Vδ1 subtype represents a large proportion of these reactive cells (143), therefore it is suspected that Vδ1 cells can contribute to antitumor immunity through a CD1-dependent pathway. Recently, it was discovered that these cells with Vδ1 TCR, specifically Vγ4Vδ1 cells, detected CD1b in transfected LCs while they producing IFN-γ after recognition (146). These cells also recognized BTN-like (BTNL) proteins, such as BTNL3 and BTNL8, which suggests that CD1b-reactive γδ T cells may respond through the engagement and bispecific combination of CD1b and BTNLs (13).

CD1c recognition has also been investigated and although it does not yet have a well-defined description, it has been shown that this isoform can be recognized by γδ T cells (147). Their involvement in detection of LCs has not yet been reported, although it is clear whether CD1c is positively regulated in LCs (144), thus hypothesizing a possible role for CD1c in γδ T cell activation. In contrast, CD1d has been extensively investigated and the molecular insights about its recognition by γδ T cells have helped us significantly to understand its participation in immune surveillance (148). Interestingly, a high expression of CD1d has been associated with a poor prognosis in leukemia (149–152), but it should be noted that Vδ3 cells can expand and respond against CD1d^+^ target cells through a CD1d-restricted reactivity and with a potent secretion of effector molecules, such as IFN-γ (60, 153). Although initial studies suggest a CD1 protein-mediated cytotoxicity, questions regarding γδ T cell subtypes and their reactivity to these ligands, in the context of leukemia, still remain.

Monomorphic MHC class I-related protein (MR1) has gained prominence after many discoveries about its regulatory role in mucosal-associated invariant T (MAIT) cell biology and its expression in cancer. This protein can mediate the recognition of folate and riboflavin derived small metabolites (154, 155). In addition, recent reports support that MR1 can present not yet defined specific tumor Ags for MR1-restricted T cells (156, 157). As expected, it was also recently established that γδ TCR recognizes this molecule (65), although direct evidence for MR1^+^ LCs detection has not yet emerged. The identification of this protein by MR1-reactive T cells may mean a new therapeutic target for cancer immunotherapy and clearly places γδ T cells on the map as a promising and important T cell population.

As discussed above, detection of LCs appears to involve many Ags and stimulatory receptors and is not driven solely by the binding of γδ TCRs to their cognate ligands, but optionally requires the involvement of additional co-receptors and targets. Other NKRs, such as DNAX accessory molecule-1 (DNAM-1), can identify their ligands, such as the polyoma virus receptor (PVR) and nectin-2 molecules, in LCs (74, 158). Although a negative role has been reported for DNAM-1 expression in leukemia (159), this co-receptor is involved in the activation of γδ T cell cytotoxicity after interaction with their ligands in leukemic blasts. This is evidenced when Vγ9Vδ2 cells kill LCs in a TCR and DNAM-1 dependent fashion, with robust secretion of perforins and granzymes (74).

Notably, Vδ1 cells can lyse LCs *via* NKp30 and NKp44, which are highly regulated *via* the synergistic signal of cytokines and TCR (66). The expression of these natural cytotoxicity receptors (NCR) is related to higher granzyme production and cytotoxicity (66). It is important to highlight that NKp30 has been proven to be crucial for Vδ1 cell-mediated antitumor response. However, NKp30 and NKp44 are bound to an as yet undetermined target (66), ignoring their classic ligands, such as B7-H6 and MLL5 that bind to NKp30 and NKp44, respectively (67), suggesting an as yet unknown additional ligand. In addition, NKp46-expressing Vδ1 cells showed higher cytotoxic activity against LCs and IFN-γ and Gzm B production, while NKp46^-^ γδ T cells showed reduced antileukemic activity (68). Despite this, the target ligand recognized by NKp46^+^ γδ T cells in LCs has not yet been demonstrated, although it is well established that cancer cells express ligands for this protein (69, 70).

## Harnessing γδ T Cells Against Leukemia: From Marrow to Blood

γδ T cells are loaded with effector weapons of great potential for cancer immunotherapy (160). Findings in recent years point to important roles for these cells, highlighting them as potential predictive biomarkers, which justifies the current focus of studies on the nature of these cells and the TME (14, 25, 161). It is important to remember that several characteristics discussed here make γδ T cells potential candidates for innovative therapies against tumors and include: (i) activation in a TCR-independent manner; (ii) the ability to recognize Ags regardless of MHC/HLA expression; (iii) effector molecules production and direct and indirect cytotoxicity potentiation against cancer cells; and (iv) their role as antigen-presenting cells (APC) that induce the proliferation of antitumor cells **(** **Figure 2** **)**.

**Figure 3:**
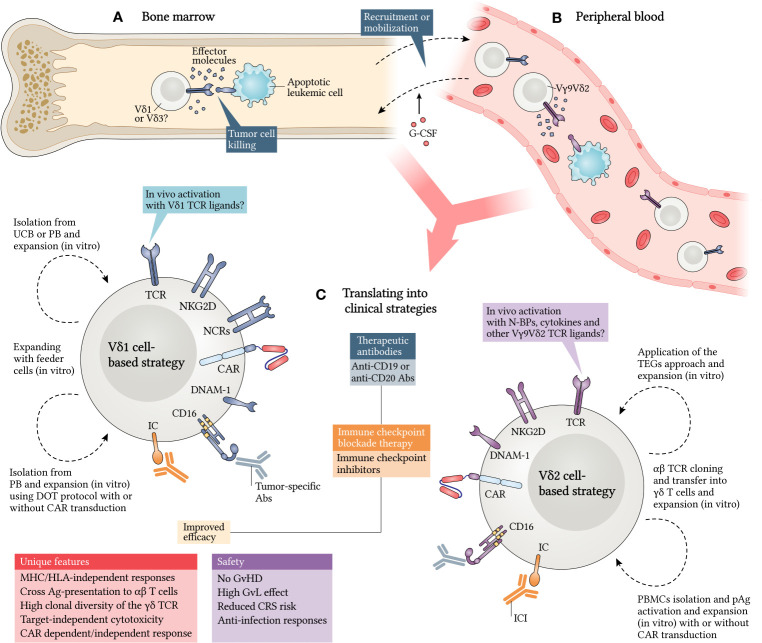
Translating γδ T cells into clinical strategies against leukemia. γδ T cells exert antitumor responses in different compartments and expressing distinct TCR patterns. Vδ1 and Vδ3 subtypes have been implicated as cytotoxic mediators in bone marrow **(A)**, while Vγ9Vδ2 cells have been shown to respond mainly in peripheral blood **(B)**. However, strategies are directed towards Vδ1 and Vδ2 subtypes, as they are the best known **(C)**. Granulocyte colony-stimulating factor (G-CSF) was shown to be a potential adjuvant to mobilize γδ T cells for peripheral blood and enrich the graft. Additionally, Vδ1 cells can be isolated from UCB or PB and expanded *in vitro* using some approaches, such as the DOT protocol, already reviewed here. *In vivo* stimulation with Vδ1 TCR ligands may be a good alternative, but it remains poorly investigated. In parallel, Vδ2 cells can be isolated from PB and activated and/or expanded *in vitro* using pAgs. A new therapeutic concept consists in the cloning and transfer of γδ TCRs into αβ T cells (TEGs) and can enhance antileukemic responses. The fact is that many of these strategies give rise to γδ T cells that express several recognition receptors and have a higher capacity to target leukemic cells (LC), which can be further improved with chimeric antigen receptor (CAR) transduction. Moreover, the use of therapeutic antibodies (Abs), such as immune checkpoint inhibitors (ICI), anti-CD19 and anti-CD20 Abs, can also provide improved efficiency in potential approaches, since γδ T cells have unique features and an attractive degree of safety for their translation into clinical trials. CRS, cytokine release syndrome; DOT, Delta One T; PB, peripheral blood; IC, immune checkpoint; TEGs, T cells engineered to express a defined γδTCRs; UCB, umbilical cord blood.

Given the high responsiveness against LCs and the absence of toxicity or alloreactivity against the host (162, 163), the application of γδ T cells in leukemia treatment may mean a new advance in cancer immunity and immunotherapy. To make this possible, several strategies for γδ T cell handling have been developed and tested and have presented interesting data **(** **Figure 3** **)**. The following subsections will focus on clinical trials and findings, as well as the activity of these cells in response to applied methods. Afterwards, we will discuss potential therapies that may specifically target γδ T cells and their subtypes, while summarizing the main approaches that are being explored to reach their clinical potential.

### Expanding γδ T Cells With pAgs, Drugs, Cytokines and Feeder Cells

Intrinsic synthesis of pAgs in cancer cells can be manipulated through pharmacological blockade mediated by aminobiphosphonates (N-BP), such as zoledronate (ZOL) and pamidronate (PAM), which interfere metabolically in the mevalonate pathway (164). The mechanism involved causes these compounds to block the enzymatic activity of farnesyl pyrophosphate synthase, which is present in this metabolic pathway. N-BP–induced interruption results in the intracellular accumulation of pAgs in cancer cells or APCs with subsequent recognition by γδ T cells and activation after cell-cell interaction (165–168). Cancer cell sensitizing with these compounds increases the tumor’s susceptibility to γδ T cell cytotoxicity, and this also applies in leukemia (132).

Some experimental evaluations took advantage of the γδ T cell recognition mechanism (directed to pAgs) to obtain a better *in vitro* or *in vivo* expansion of these lymphocytes and test their therapeutic efficacy. To date, these approaches have focused on ZOL, (E)-4-Hydroxy-3-methyl-but-2-enyl pyrophosphate (HMB-PP) and synthetic pAgs, such as bromohydrin pyrophosphate (BrHPP) (169, 170). These compounds are generally administered in combination with low cytokine doses such as IFNs, IL-2, IL-12, IL-15, IL-18 and IL-21. In addition, these approaches can induce an antitumor phenotype and the pronounced expression of associated receptors (171–176).

Vγ9Vδ2 cell expansion has become more accessible because, in addition to being the most prevalent subtype in PB (55, 56), it can also recognize a diversity of relatively well-defined target molecules (177). When these cells are treated with ZOL + IL-2 + IFN type I, their cytotoxic activity is increased and Vγ9Vδ2 cells may be able to efficiently destroy lymphoid and myeloid lineage LCs, as proposed by Watanabe et al. (171). In their study, γδ T cells were generated *in vitro* with ZOL + IL-2 for 14 days, and after this period they were activated with IFN type I for up to 3 days. Thus, the resultant γδ T cells were well expanded in the culture and showed a significant expression of CD69, TNF-related apoptosis-inducing ligand (TRAIL), IFN-γ and TNF, which suggests the acquisition of an activated phenotype and antileukemic reactivity.

In the same vein, sensitization with ZOL + Imatinib has also been shown to increase the cytotoxic synapse between Vγ9Vδ2 cells and LCs (178). Initially, Imatinib resistant or sensitive LCs had low susceptibility to γδ T cells, but *in vitro* treatment with ZOL + Imatinib was able to reverse this situation. The lysis of these LCs was mediated by TCR, NKG2D, TRAIL and perforins. This high cytotoxicity was dependent on ZOL, since it was observed that Vγ9Vδ2 cells exerted low antitumor activity that was slightly increased after sensitization of LCs. To validate these findings, it was further demonstrated that when Vγ9Vδ2 cells, ZOL and IL-2 are infused in a leukemia mouse model, they mediate tumor regression *in vivo* and confer greater survival in these mice (178).

The ability of N-BPs to invigorate exhausted Vγ9Vδ2 cells has also been reported in other investigations (179, 180) and appears to be a promising alternative for their use, given that a higher exhausted γδ T cell frequency has also observed in leukemia (27, 125). It is important to note that, in this context, these cells exhibit a low expression of CD107a, FcγRIII (CD16), IFN-γ and TNF, while B and T lymphocyte attenuator (BTLA), LAG3 and PD1 proteins are more highly regulated on their cell surface (179, 180). When cultured with allogeneic LCs, these lymphocytes had low cytotoxic activity, while γδ T cells from healthy patients responded efficiently (179). Notably, when Vγ9Vδ2 cells that were considered dysfunctional were cultured *ex vivo* with mature monocyte-derived dendritic cells (Mo-DC) and N-BPs for 8 days without the presence of LCs, the observed functional impairments could be reversed (179).

Ibrutinib has also been shown to activate γδ T cells against LCs, since Weerdt et al. reported that it was able to induce an antitumor phenotype (180). In their study, allogeneic and autologous γδ T cells were cultured with LCs. As already seen, γδ T cells from patients with leukemia proved to be dysfunctional in terms of cytokine production and cytotoxicity, while those from healthy patients had a strong antitumor activity (179). When Vγ9Vδ2 cells from both cases are treated with Ibrutinib, an effector Th1 phenotype and memory cells are induced. Overall, their antitumor properties can be recovered after *ex vivo* stimulation and after treatment with Ibrutinib, which binds to the IL-2–inducible T cell kinase molecule and promotes activation against LCs (180).

Other investigations have presented a new alternative: the combination of IL-15 plus N-BPs or pAgs promotes significantly greater expansion, high cytotoxicity and a more pronounced Th1 phenotype in γδ T cells, when compared to expansion methods using only IL-2 (174, 181, 182). IL-15 is a powerful growth factor for γδ T cells (183, 184) and can synergize with other molecules and enhance the antileukemic capacity of these cells **(** **Figure 2** **)**, as we will highlight below.

The *ex vivo* tests carried out by Van Acker et al. (174) demonstrated that the administration of IL-15 + isopentenyl pyrophosphate (IPP) is able to improve γδ T cell cytotoxicity against LCs. In contrast, γδ T cells stimulated with IL-2 + IPP were more likely to deviate to a Th2 and Th17-like response phenotype when interacting with LCs (174). It is important to highlight that stimulation by IL-15 promoted a more robust IFN-γ and TNF secretion when compared to IL-2 stimulation. In addition, culturing these lymphocytes with IL-2, IL-15 and ZOL for 14 days critically enhanced the expansion rates to almost 1000-fold the total yield of viable cells, which showed a 590-fold increase in the γδ T cells cultured only with IL-2 + ZOL (174).

Interestingly, when IL-2 + IL-15 and ZOL are administered to γδ T cells isolated from patients with leukemia, during 14 days of culture, they assume different phenotypic states. Most of them may exhibit an effector memory phenotype (CD45RA^-^ CD27^-^), followed by a central memory phenotype (CD45RA^-^ CD27^+^) (174). In addition, positive regulation of CD56, CD80 and CD86 is also provided (174), suggesting that, in addition to exerting strong antileukemic activity, these cells may also act as APCs and improve the antitumor responses.

Vγ9Vδ2 cell expansion using IL-2 may not even promote satisfactory proliferative rates; however, it is clear that the synergism between IL-2 and IL-15 confers a substantial increase in an inflammatory profile (174, 181), as these cytokines promote a higher transcription factor T-bet expression (181), which in turn, is related to greater cytotoxicity. In addition, the advantage of γδ T cells expanded with IL-2 + IL-15 can be maintained under one of TME’s hallmarks *in vivo*, namely hypoxia (181). In fact, a striking feature of the leukemic microenvironment is the low partial pressure of oxygen that favors the tumor-associated immunosuppressive pathways, while at the same time promoting expansion of LCs (185). In this context, the persistence of γδ T cells in hypoxia further demonstrates their clinical importance.

Alternatively, the combination of ZOL, IL-2 + IL-18 also promotes the proliferation of effector cells (186, 187) since IL-18 is an important inducer of IFN-γ secretion (188). Given this, it has been reported that this cytokine indirectly induces the expansion of γδ T cells. Tsuda et al. (186) showed that Vγ9Vδ2 cells are efficiently expanded in response to ZOL, IL-2 + IL-18, but in a CD56^bright^ CD11c^+^ NK-like cell dependent fashion (187). Many studies have reported that the involvement of NK-like cells in the proliferation of γδ T cells implies greater expansion efficiency when compared to methods using dendritic cells (DC) or monocytes (187, 189–191). These findings suggest an approach targeted at feeder cells that may be responsible for γδ T cell clonal proliferation in different methods *in vitro* and, perhaps, *in vivo*.

IL-18 can also directly support γδ T cell expansion, even in the absence of feeder cells (192). When Vγ9Vδ2 cells are treated only with ZOL, there is a delay in their *in vitro* expansion, as prolonged exposure subjects these cells to acute ZOL toxicity (193). However, when IL-18 combined with geranylgeranyl pyrophosphate (GGPP) is added, the proliferative capacity is restored by inhibiting the toxic effects of ZOL, which allows a substantial expansion of viable γδ T cells to occur. IL-18 + GGPP also were able to activate γδ T cells, exhibiting a central memory or effector memory phenotype and with higher IFN-γ production and CD56 expression (192).

In a subsequent study, treatment with ZOL + IL-2 and culture with Mo-DCs stimulated an activated phenotype in γδ T cells. In this context, immature Mo-DCs have been shown to have a particularly higher capacity to intensify γδ T cell cytotoxicity against LCs, whether in autologous or allogeneic condition (194). Furthermore, IL-15 producing DCs isolated from healthy patients and patients with leukemia (in remission) can potentiate γδ T cell cytotoxicity *in vitro* (182). These DCs induced NKp30, CD16, CD80 and CD86 expression in γδ T cells in an IL-15 dependent manner. This methodology was able to produce γδ T cells with higher expression of co-stimulatory molecules and low expression of inhibitory proteins. In addition, stimulation with DCs + IPP + allogeneic LCs led to high IFN-γ secretion and strong antitumor activity (182).

Deniger et al. (162) demonstrate a new strategy that involves the use of artificial APCs (aAPCs) derived from the K562 leukemic lineage. These feeder cells were modified to express molecules, such as CD19, CD64, CD86, 4-1BBL and IL-15, on their membrane surface. When γδ T cells are cultured with aAPCs + IL-2 + IL-21, there is a remarkably robust 4900 ± 1700-fold polyclonal expansion (162). Most of these cells expressed different γδ TCR domains. Resultant γδ T cells also were able to kill LCs *via* TCR, NKG2D and DNAM-1 (162).

In the same vein, Cho et al. (175) used CD80^+^, CD83L^+^ and 4-1BBL^+^ aAPCs. At low IL-2 concentrations, these co-stimulatory molecules promoted a remarkable Vγ9Vδ2 cell expansion that secreted higher levels of IFN-γ and TNF (175). Notwithstanding, there was no significant proliferation rate (106-fold increase) when compared to the hefty increase observed in the previous study (162). Triple co-stimulation with these molecules induced not only the high IFN-γ and TNF production, but also the positive regulation of a range of other molecules such as IL-2, IL-6, perforins, Gzm A and Fas ligand (FasL) (175). Most importantly, the expanded cells exhibited a terminal effector phenotype (CD27^low^ CD45RA^high^), followed by an effector memory phenotype (175).

Unlike most of the investigations discussed above, other studies have focused on γδ T cells that express the Vδ1 TCR chain. Substantial evidence has demonstrated the ability of this subtype to kill LCs, as already reviewed. Unlike the Vγ9Vδ2 subtype, these cells do not show susceptibility to activation-induced cell death (AICD), which has been reported in several experimental trials (125, 195, 196). These cells can also exercise immune surveillance for long periods, favoring the longevity of cancer immunity (197–199). Several unique attributes have been discovered that particularly place Vδ1 cells as attractive targets in antileukemic therapies. So far, a few studies have emerged that have sought to translate the functional role of these lymphocytes and their applicability, as we will highlight below.

Siegers et al. (30) developed an *in vitro* expansion protocol that enabled the proliferation of γδ T cells isolated from PB after treatment with lectin-based compounds named Concanavalin-A (Con-A). Thus, it was possible to expand Vδ1 cells in a greater proportion than the Vγ9Vδ2 subtype when Con-A was combined with IL-2 + IL-4. The low Vγ9Vδ2 cell proportion was motivated by the period of exposure to Con-A, which induced AICD in these lymphocytes (30). Noteworthy, the resulting Vδ1 cells exerted an efficient cytotoxic activity against LCs through TCR, NKG2D, CD56 and FasL (30).

Subsequently, proof-of-concept studies were performed on leukemia xenograft models using a newly established cell generation protocol called Delta One T (DOT), which was designed by Almeida et al. (31). Specifically, this clinical-grade protocol consists of two steps. First, γδ T cells are isolated from PB of healthy donors or patients with leukemia using magnetic beads and are cultured *in vitro* for 14 days. During this time, these lymphocytes are expanded using a combination of molecules, such as IFN-γ, IL-1β, IL-4 + IL-21, in association with anti-CD3 antibodies (Abs). Then, the expanded cells are transferred to a new culture medium, where they are restimulated by anti-CD3 combined with IL-15 and IFN-γ for another 7 days (31). Overall, this is a 3-week protocol that involves γδ TCR and cytokine stimulation that can accomplish its goals efficiently.

When γδ T cells were submitted to the DOT protocol, expansion was obtained with rates greater than 1000-fold, thus allowing the viable and efficient proliferation of highly cytotoxic cells. It is noteworthy that, with this cell proportion rate, Vδ1 cells, which are generally less frequent in the blood (55, 56), expand from less than 0.5% of all circulating T cells to more than 70% (25, 31). Notably, Vδ1 cells with high expression of NKp30, NKp44, DNAM-1 and 2B4 are also provided, all well established as key-receptors in antileukemic responses (66, 74). These lymphocytes do not regulate inhibitory proteins on their membrane surface, even after 3 weeks of continuous stimulation. In addition, many cell adhesion molecules and chemokine receptors are positively regulated, while these lymphocytes can kill autologous and allogeneic LCs *in vivo*, and ignoring normal cells (31).

Finally, the same protocol was tested by Lorenzo et al. (200), in which γδ T cells from PB were reinforced using a range of stimulatory molecules (31). While the previous study sought to mobilize Vδ1 cells against a CLL xenograft model (31), the latter work applied the DOT protocol to an AML xenograft model (200). It is important to highlight that in both cases there was an efficient regression of tumors, and this increased mice survival (31, 200). In addition, γδ T cells avoided systemic metastasis of LCs (31). They exerted their antileukemic activity against AML blasts in a partially TCR-dependent manner, while they depended on the B7-H6 expression (200), which binds to NKp30 (67).

### Blocking Immune Checkpoints in γδ T Cells and Leukemic Cells

Although they are potent, γδ T cell antitumor responses can be regulated by immune checkpoints (IC). Many inhibitory proteins, such as PD1, CTLA4, LAG3, BTLA, T cell immunoreceptor with Ig and ITIM domains (TIGIT) and T cell immunoglobulin and mucin domain-containing protein 3 (TIM3), are key mediators in inflammatory regression and cell suppression, in the context of the TME (93, 201, 202). Generally, these molecular interactions can act synergistically with the infiltration of suppressive cells that support tumor evasion through the establishment of a strongly tolerogenic environment (26, 76). However, recent advances in cancer immunotherapy using monoclonal Abs (mAbs) targeting ICs, the immune checkpoint inhibitors (ICI), have shown that combinatorial blocking of proteins, such as PD1 and PD-L1, can restore cellular functions and reestablish antitumor activity (203, 204).

The mechanisms of γδ T cell regulation mediated by ICs are diverse and poorly understood, but seemingly unified by the fact that these receptors functionally complement each other and ensure the adjustment of the immune response. PD1 and BTLA are the most potent ICs shown to suppress γδ T cell cytotoxicity in cancer (205, 206). Although CTLA4 expression has not been consistently assessed, it is known that this molecule is rarely expressed in activated γδ T cells (207, 208). Importantly, the expression of these ICs may vary between γδ T cell subtypes, where, for example, Vδ1 cells exhibit higher PD1 expression than their Vγ9Vδ2 counterpart (209).

Early after activation, when the γδ TCRs find their cognate ligands, γδ T cells begin to rapidly display many of these ICs on the cell surface (205, 207). Collectively, the expression of these proteins is low or stable, but temporary, and is sufficient to reduce cytokine production, proliferation and survival of γδ T cells (205, 206, 208, 210, 211). These changes can also be observed in leukemia, as γδ T cells increase the expression of PD1, CTLA4 and BTLA, while LCs strongly regulate the expression of their ligands, such as PD-L1, CD80 and/or CD86, and herpesvirus-entry mediator (HVEM), respectively (212). This represents an important barrier, as these molecules can prevent the efficient activation of γδ T cells and the associated antitumor response. Blocking the expression of these inhibitory receptors through the use of ICIs may be an interesting alternative to reverse the state of anergy and/or cell exhaustion.

The influence of ICIs on γδ T cells and their potential impact on the associated cytotoxic activity, in the context of the leukemic microenvironment, has not yet been characterized and is, therefore, an open question. Despite this, PD1 has been shown to negatively regulate Vγ9Vδ2 cell responses, while the addition of ZOL + anti–PD-L1 was able to bypass the inhibitory signals and promote γδ T cell reactivation against LCs in a TCR-dependent fashion (205). Therefore, this discovery allows us to suggest that γδ TCR-mediated activation is capable of overcoming the inhibitory effects of the PD1/PD-L1 pathway, since the application of ICIs plus ZOL, which is a strong Vγ9Vδ2 TCR stimulator, apparently synergizes the activation of γδ T cells and restores their tumor reactivity (205).

Notably, Hoeres et al. (213) demonstrated that although PD1 signaling can modulate the production of IFN-γ in leukemia-reactive γδ T cells, its additional blockage and stimulation with ZOL can increase the production of this cytokine. Although it did not show a significant effect on the destruction of LCs by γδ T cells, the action of anti-PD1 + ZOL in these lymphocytes was able to induce high IFN-γ secretion, which is a potent inflammatory and antitumor factor (213). As noted, cytokine secretion, such as IFN-γ, can be negatively regulated, and we can infer from this study that the application of ICIs potentially reverses this suppressive condition and is able to stimulate the triggering of an antitumor response.

In addition to PD1/PD-L1, other inhibitory proteins are highly regulated in LCs (i.e., CTLA4, BTLA, TIGIT, TIM3 and LAG3) and their effects on γδ T cells have not yet been fully investigated (26, 159, 214–216). However, previous studies have shown that some of these receptors have great potential for deregulating their antitumor activity, reflecting in cytokine production (213, 217). Nonetheless, evaluating these components before proceeding to a clinical application is important, since these molecules most likely prevent the efficient killing of LCs. This is one of several mechanisms of tumor escape that are commonly observed in recent and innovative treatment modalities, and which also include the chimeric antigen receptor (CAR) T cell therapy (218).

### Focusing on γδ T Cell-Engager Molecules in the Leukemic Microenvironment

#### Antibodies Direct γδ T Cells Against LCs

As we have shown herein, data from *in vitro* experiments and mouse models unequivocally demonstrate the potential of γδ T cells against leukemia. Knowledge obtained regarding the many signals that regulate their activation and the tumor resistance underlying γδ T cells offers additional approaches that, in addition to inducing an activated status, a (poly)clonal expansion or a more pronounced Th1 phenotype, may also allow more specific targeting against the tumor. Improving γδ T cell efficiency against LCs, however, requires strategies based on their cytotoxic nature, which include, for example, antibody-dependent cell cytotoxicity (ADCC) (75, 219). Therefore, this implies a role for CD16, mAbs and bispecific antibodies (bsAbs) that bind to their respective target antigens.

CD16-mediated ADCC plays an important role in tumor destruction. For this to occur, CD16 must bind to the constant fraction of Abs IgG, thus constituting an optional axis in target cell killing. γδ T cells constitute the major blood T cell population that expresses CD16 (220, 221), although this expression is variable (222). Given this, the potential engagement of therapeutic Abs with the product of Vγ9Vδ2 cells can provide an efficient alternative against LCs (223). Several studies have shown that γδ T cells mediate leukemic regression *via* a CD16-dependent pathway (136, 223–226), in particular the Vγ9Vδ2 subtype, which positively regulates CD16 and TNF expression when stimulated with pAgs (227).

mAbs-coated LCs are efficiently destroyed by CD16^+^ γδ T cells *via* ADCC and these lymphocytes subsequently exhibit APC functions and activate αβ T cells, apparently through the tumor Ags presentation by MHC class II (228). It has been shown that the application of therapeutic CD20-targeting Abs, such as Rituximab (RTX), improves the antileukemic effect of these lymphocytes through tumor destruction by ADCC *in vitro*. This leads γδ T cells to secrete high levels of IFN-γ, perforins and CCL5 (219). In addition, BrHPP implementation potentiates the RTX bioactivity and consequently also increases γδ T cell cytotoxicity against CD20^+^ LCs *in vitro* and *in vivo* (75).

When peripheral blood mononuclear cells (PBMC) are stimulated with ZOL + IL-2 *ex vivo* and then cultured with LCs and Obinutuzumab (anti-CD20), it is observed that γδ T cells perform ADCC more efficiently than NK cells (223). Most importantly, the cytotoxicity of these lymphocytes cultured with Obinutuzumab is more potent compared to other tested mAbs, such as RTX. This view was reinforced when LCs treated with Obinutuzumab were substantially lysed in a CD16-dependent manner (223).

Benyamine et al. (136) demonstrated that BTN3A-targeting mAbs (anti-BTN3A 20.1) sensitize LCs and act indirectly in tumor destruction. This is due to the anti-BTN3A Abs binding in three different target molecules: BTN3A1, BTN3A2 and BTN3A3. The combination of these mAbs with γδ T cells and the subsequent infusion in a leukemia murine model was able to decrease the leukemic load in the PB and BM, increasing survival in these mice (136). Taken together, these data create the expectation that targeting mAbs to BTN proteins can be potentially useful in new therapeutic approaches.

Like most other surface molecules expressed in LCs, CD19 is also a potential target to be considered. When LCs are incubated with γδ T cells and modified anti-CD19 Abs (Ab 4G7SDIE), a significant increase in the degranulation marker CD107a is observed, as well as the strong IFN-γ and TNF production (224). In addition, the adoption of bsAbs targeting CD19/CD16 (bsAbs N19-C16) is also able to increase the expression of these inflammatory molecules (224). Interestingly, bsAbs targeting CD19/CD3 (bsAbs N19-CU) also strongly activated γδ T cells and, unlike the other previously tested Abs, mediated the lysis of LCs (224). It should be noted that the use of Abs modified to have a triple specificity to CD16 and CD19 (triplebody SPM-1) was also able to activate these lymphocytes against CD19^+^ target cells, which was evidenced by the expression of antitumor mediators (225).

The projection of a bsAbs targeting the Vγ9 TCR chain and CD123 (anti-TRGV9/CD123 engager) was also able to recruit γδ T cells against AML blasts (229). This engagement induced its activation and cytotoxicity against endogenous LCs, as evidenced by CD69, CD25 and Gzm B positive regulation. Interestingly, these activated γδ T cells exhibited a low secretion of IL-6 and IL-10, which are cytokines that are highly related to cytokine release syndrome (CRS) in patients undergoing αβ T cell-based therapies (229–231). The efficacy of this approach is evidenced when anti-Vγ9/CD123 directed γδ T cells were infused into a leukemia mouse model and controlled the leukemic proliferation in different compartments in these mice (229).

Finally, it has been shown that CD1d is also an attractive target. A recent study showed that CD1d specific single domain Abs can guide γδ T cells (226). These engagers were able to mobilize and activate these lymphocytes against autologous LCs from patients with CLL. This allowed γδ T cells to produce many inflammatory molecules and maintain their pAgs reactivity (226). Taken together, the many studies reviewed here allow us to suggest that the therapeutic application of Abs can be improved with the use of N-BPs that enhance γδ T cell activation. However, their therapeutic application against leukemia still needs more detailed investigation.

#### γδ T Cells Expressing CARs

While the application of therapeutic Abs has significantly increased the effectiveness of leukemia treatments, other approaches are also emerging with promising healing potential. Current advances in genetic engineering enable CAR transduction in NK cells, macrophages and T cells, thus offering new horizons for cell therapy, although this has been primarily focused on conventional αβ T cells (232, 233). In this context, γδ T cells are also undergoing a number of improvements in order to enhance their antitumor capacities.

The fact is that γδ T cells can be redirected with CARs against surface molecules expressed by LCs (234). Their unique innate properties and their high capacity for tumor sensing and killing place them in an interesting position in potential approaches against leukemia. CAR γδ T cells can offer a triple activity because, for example, they can recognize LCs (i) through the direct engagement of γδ TCR to their cognate ligand, (ii) through NKRs and their associated ligands, or (iii) through CAR specificity to the target antigen **(** **Figure 3** **)** (234, 235). Besides this, their APC functions (211) may allow the prolongation of immune response in the TME (228), since the CAR acquisition preserves the ability of γδ T cells to present tumor Ags (235).

The applicability of these genetically modified T cells has been established by some of the previous studies that evaluated the viability of viral transduction (236, 237) or electroporation (238) of the CAR. Rischer et al. (236) demonstrated for the first time that Vγ9Vδ2 cells can be efficiently transduced with CAR genes. Their study also showed that γδ T cells expressing anti-CD19 CARs destroy CD19^+^ LCs and produce high levels of IFN-γ in a target-dependent fashion (236). Subsequently, Deniger et al. (238) showed that the introduction of CAR by electroporation in PB-derived γδ T cells is able to produce polyclonal CAR T cells that express Vδ1, Vδ2 and Vδ3 TCR chains (238). For this to happen, approaches already reviewed here were used (162).

Noteworthy, one study demonstrated that CAR γδ T cells adopt a highly activated, but not exhausted, phenotype, as highlighted by the low regulation of CD57 (238). In addition, these lymphocytes tend to assume distinct phenotypic states of effector memory, while positively regulating homing molecules. Specifically, these homing receptors included CXCR4, a molecule associated with migration to BM, as well as CD62L and CCR7, which are linked to migration to lymph nodes (238). This is encouraging since BM and lymph nodes are sites of high tumor growth in acute and chronic leukemias (1, 4, 26, 78, 239–241).

Surprisingly, it has also been confirmed that CAR γδ T cells recognize and kill LCs in BM regardless of the CD19 target. Rozenbaum et al. (242) recently showed that these modified lymphocytes have high IFN-γ production and reactivity to CD19^+/-^ LCs *in vitro*, which was even enhanced with the addition of ZOL. To investigate *in vivo* efficacy, the authors injected CAR γδ T cells in a leukemia mouse model. Although it did not induce a complete remission, the infusion of these cells led to a drastic reduction in the leukemic burden in the BM of these mice, which was even more pronounced when ZOL was administered (242).

These studies demonstrate that the production of CAR γδ T cells is viable and supports the high effectiveness of these lymphocytes against many malignancies, especially in leukemias. In contrast to conventional CAR T cell therapy, approaches based on γδ T cells can overcome several currently reported limitations, such as modulation of tumor antigen expression (242, 243) and CRS (229–231).

#### How About Molecular Switching of TCRs?

One interesting strategy for targeting lymphocytes against the tumor is to design γδ T cells with αβ TCRs or to design αβ T cells with γδ TCRs (244). This therapeutic concept has great potential for combining some unique γδ T cell properties, such as the rapid responsiveness to the tumor, the expression of individual molecules, and the absence of alloreactivity, with the high proliferative capacity and specific reactivity of conventional αβ T cells. Combining these unique aspects through TCR transduction leads us to expect that the resulting antileukemic responses will be long-lasting and based on immunological memory.

This new concept of modified T cells, named T cells engineered with defined γδ TCRs (TEG), was adopted in some studies that showed that TEGs kill LCs *in vitro* and *in vivo* models (245). TEGs tend to deregulate the intrinsic αβ TCR expression in their membrane surface, avoiding the graft-*vs*-host disease (GvHD) (245, 246). In addition, CD4^+^ TEGs retain their ability to induce a complete maturation of DCs, and stimulation with PAM can potentiate the cytotoxicity of CD8^+^ or CD4^+^ TEGs since it promotes higher production of inflammatory molecules, such as IFN-γ, TNF, and IL- 2, *in vivo* (245).

Similar results were obtained when TEGs cultured with LCs reduced the tumor *in vitro* (247). In addition, the infusion of TEGs plus IL-2 + PAM in an AML murine model enabled reactivity directed to LCs without affecting the healthy hematopoietic compartment and without being influenced by the TME, when inserted into mice that expressed IL-3, granulocyte-macrophage colony-stimulating factor (GM-CSF), and stem cell factor (SCF) (247), which are molecules that support tumor growth *in vivo* (248). Therefore, TEGs demonstrated efficiency in reducing the tumor in xenograft models with minimal alloreactivity, which stimulated the projection of a robust manufacturing procedure of TEGs that were validated under good manufacturing practice (GMP) conditions (244, 249).

Finally, γδ T cells transduced with αβ TCR plus CD4 and CD8 co-receptors showed high antitumor activity against LCs (250). As similarly observed in TEGs, transduction of αβ TCR induced a low expression of endogenous γδ TCR. In addition, modified CD8^+^ or CD4^+^ γδ T cells expressed high levels of IFN-γ and IL-4, although IFN-γ production was more pronounced in CD8^+^ cells. Most importantly, these transduced cells were able to kill LCs *in vitro*, although CD8^+^ γδ T cells have shown more efficiency than CD4^+^ cells (250, 251). This evidence supports the important role of γδ T cells in TCR gene transfer-based approaches while suggesting an improved antileukemic capacity when TCR transduction is combined with co-receptors, in particular, with the CD8 protein.

### Converting γδ T Cells Into Living Drugs

#### Source, Isolation and Pre-Activation

γδ T cells and their subtypes are present in several tissues, but the ideal source for obtaining all these lymphocytes is still being determined. Despite this, therapeutic γδ T cells for infusion can be obtained from peripheral blood (252, 253) or umbilical cord blood (UCB) (254, 255). It is important to note that the frequency of γδ T cells varies between 5-10% of peripheral blood T cells (51, 52), while they constitute <1% of T cells in UCB (254). The functional differences between γδ T cell subtypes in these sources are not yet clear, but it is already established that while the subtype expressing Vγ9Vδ2 TCR predominates in PB (51, 55, 56), polyclonal γδ T cells expressing the Vδ1 TCR domain predominate in UCB (52, 256, 257).

γδ T cell expansion from PB is a well-established method and is usually adopted in clinical and experimental trials. For isolation of these lymphocytes, the starting material is the product of leukapheresis, which can be initially enriched through stimuli with several soluble factors (e.g., cytokines and N-BPs) and later undergoes removal of αβ T cells and CD19^+^ B cells through the use of magnetic beads, depletion or separation kits (optionally maintaining NK cells) (169, 252, 253). Since increasing the γδ T cell product from leukapheresis can further improve its therapeutic handling, adopting the use of molecules as the granulocyte colony-stimulating factor (G-CSF) may mobilize a large amount of antileukemic γδ T cells for peripheral blood, as shown in several studies (258–262).

Alternatively, physical exercise and the consequent systemic activation of β-adrenergic receptors (β-AR), immediately before PBMC isolation, has been shown to substantially increase mobilization for PB, *ex vivo* expansion and antitumor capacity. In their study, Baker et al. (263) showed that the practice of physical exercises can predict the expansion potential of γδ T cells, which is mobilized in a β-AR type 2 dependent fashion. Therefore, patients with high levels of physical activity mobilized γδ T cells that expanded *ex vivo* in much higher percentages compared to blood at rest when stimulated with IL-2 + ZOL for 14 days (263). These cells had higher expression of CD56 and NKG2D and showed high cytotoxicity against LCs *in vitro*.

On the other hand, γδ T cell isolation from UCB is still poorly investigated and so far, it has not been the target of cell expansion protocols in clinical trials. Berglund et al. (264) showed that it is possible to expand γδ T cells derived from UCB *in vitro*. The authors developed an expansion protocol based on the application of ZOL + IL-2 in culture for 14 days. This promotes the growth of Vγ9Vδ2 cells that mostly adopt a central memory phenotype and secrete higher levels of IL-1β, IL-2 and IL-8 (264). In general, the acquisition and handling of UCB-derived γδ T cells still need to be investigated more fully. Some factors, such as the low frequency of Vγ9Vδ2 cells (more easily expanded *in vitro*) in UCB and the poorly defined phenotypic diversity in this environment, make handling more limited (254). The approaches discussed here are viable targets for adoptive cell therapy because they also serve as adequate and economical adjuvants for hematopoietic stem cell transplantation (HSCT) (263, 264).

It is not clear whether pre-activation with ZOL + IL-2 can trigger the total antitumor capacity of γδ T cells. However, many *in vitro* approaches that use other molecules, such as IL-15, have demonstrated greater potential in stimulating the activation of these lymphocytes. As already reviewed, IL-15 associated with pAgs promotes high cytotoxicity in γδ T cells, which is evidenced by the high T-bet expression (181). In addition, the combined use of IL-2 + IL-15 can provide γδ T cells with antileukemic properties (174, 181, 182) even in hypoxia (181).

A mix of cytokines combined with Abs can also promote a pre-activated state in γδ T cells, as evidenced in studies using the DOT protocol. Notably, the use of IFN-γ, IL-1β, IL-4, IL-15, and IL-21 with anti-CD3 Abs positively regulates many NKRs, while ICs, such as PD1, CTLA4 and CD94/NK group 2 member A (NKG2A), are negatively regulated on the cell surface (31, 200). In addition, many homing receptors, such as signal-regulatory protein alpha (SIRPα), integrin-β7, CD31, CD56, CD96 and intercellular adhesion molecule 1 (ICAM-1), are expressed, as well as chemokine receptors, such as CXCR3, CCR6 and CX3C chemokine receptor 1 (CX3CR1) (31). Noteworthy, the junction of these cytokines promotes γδ T cells with APC functions and a higher potential to migrate and recirculate between blood and tissues (31, 174). Therefore, pre-activation using these approaches may lead to better crosstalk with other cytotoxic cells (e.g., NK) or LCs in different compartments (265).

#### The HSCT Questions

The functional importance of γδ T cells in HSCT has received enormous attention after many years of research. The fact is that the frequency of these lymphocytes may fluctuate between treated and untreated individuals, either during chemotherapy (27, 266) or after HSCT (267–273), implying relevant roles for γδ T cells in the patient’s recovery (274). Several initial reports have shown that αβ TCR depleted allogeneic HSTC (allo-HSCT) was able to increase disease-free survival (2-5 years) after transplantation (267, 268, 273). Notably, this was correlated with a high γδ T cell frequency circulating in the PB and mediating the graft-*vs*-leukemia (GvL) effect (267). The Vδ1 subtype represented the highest proportion of these cells in the blood of patients (267, 273, 275).

Given that γδ TCRs are not restricted to HLA expression, the triggering of the GvHD effect is less likely, since tumor detection depends on more ubiquitous targets (273, 276). Therefore, the high frequency of these cells contributes to the restoration of the hematopoietic niche and is related to antileukemic responses (273); although this is not their only contribution to the success of HSCT. Higher γδ T cell percentages and a lower incidence of infection was been observed in many patients after HSCT, indicating protective roles in fungal, bacterial and viral infections (268, 273, 276). This made it possible to increase survival in patients with a high frequency of these cells when compared to patients with low or normal counts (277).

Cytomegalovirus (CMV) infection and its reactivation is a major concern after HSCT and, notably, γδ T cells can be essential effectors in controlling viral expansion. Knight et al. (278) reported for the first time that Vδ1 and Vδ3 cells expand as a result of an active response against CMV in patients after allo-HSCT; although there were previous data that showed that these subtypes expand in CMV infection in immunocompetent individuals (275, 276, 279). Interestingly, CMV reactivation after allo-HSCT mobilized these non-Vδ2 subtypes against infected cells and against LCs *in vivo* (280). This is intriguing and leads us to infer that the reactivation of CMV after HSCT can benefit patients with leukemia, as it impacts the incidence of disease recurrence (281).

Epstein-Barr virus (EBV) infection is also a problem. Farnaut et al. (282) showed that EBV infection resulted in a significant Vδ1 cell expansion in a patient with ALL transplanted with UCB, which represented more than 80% of the total circulating γδ T cells. One year after transplantation, these cells were highly differentiated and exhibit CD57 and CD8 expression while minimally expressing the BTLA protein (282). These data suggest a strongly adaptive response from Vδ1 and Vδ3 cells that possibly improves the efficacy of allografts (269).

Overall, the graft enriched with γδ T cells provides a lower relapse incidence during immune reconstitution after HSCT (274). This is evidenced when patients with low frequencies of these lymphocytes have a high rate of death from relapse (283). In addition, γδ T cell innate and adaptive responses can also prevent the occurrence of infections after HSCT (269, 284, 285). Finally, their functional plasticity can assist in immunological tolerance to the graft and avoid GvHD, as evidenced in many studies (258, 260). Therefore, the data highlighted here position γδ T cells as potential targets in applications aimed at improving clinical results after HSCT, since they induce a potent GvL effect in the absence of GvHD.

## The State-of-the-Art for Clinical Trials

Although promising, γδ T cells have not yet been fully translated into clinical research that targets leukemia. Although clinical studies carried out over two decades have shown that γδ T cells have low toxicity and reactivity against the host (274), the clinical efficacy of adoptive therapy with γδ T cells has not been consistently reported **(** **Table 2** **)**. *In vivo* stimulation, that is, the activation of autologous γδ T cells using N-BPs + IL-2, induced few measurable responses in patients with leukemia. Wilhelm et al. (286) included 4 patients with CLL in a clinical study based on PAM + IL-2 *in vivo* infusion. None of the 4 patients were able to obtain objective or complete responses, which was also evidenced by the low expansion of endogenous γδ T cells *in vitro* when isolated from these patients.

**Table 2:** Executed clinical trials with γδ T cell-based strategies.

Leukemia subtype	*N* included	Interventions	Objective response	Complete response	Ref.
***In vivo* stimulation (autologous)**					
CLL	4	PAM and IL-2	0/4	0/4	(276)
AML	8	ZOL and IL-2	2/8	0/8	(277)
ALL and AML	43	ZOL	ND	ND	(261)
ALL, AML and MPAL	46	ZOL after allo-HSCT depleted for αβ T cells/CD19^+^ B cells	ND	ND	(262)
***Ex vivo* expansion (donor γδ T cells)**					
ALL, AML and CLL	74	Allo-HSCT depleted for αβ T cells	43/74	25/43	(257)
ALL and AML	153	Allo-HSCT depleted for αβ T cells	100/153	36/153	(258)
AML and SPL	2	ZOL and IL-2 after CD4/CD8 depleted haplo-PBMC	2/2	2/2	(278)

ALL, acute lymphoblastic leukemia; AML, acute myeloid leukemia; allo-HSCT, allogeneic hematopoietic stem cell transplantation; CLL, chronic lymphocytic leukemia; haplo, haploidentical; IL, interleukin; MPAL, mixed phenotype acute leukemia; ND, not determined; PAM, pamidronate; PBMC, peripheral blood mononuclear cell; SPL, secondary plasma cell leukemia; ZOL, zoledronate.

Kunzmann et al. (287) evaluated stimulation with ZOL + IL-2 in several tumors. In this clinical trial, 8 patients with AML were included. Only 2 of them had an objective response, and they achieved a partial remission. Notably, ZOL infusion in pediatric patients with acute leukemia after HSCT depleted for αβ TCR and CD19^+^ B cells prolonged the disease-free survival in these patients, since it was associated with high numbers of circulating γδ T cells (271). This was also reported in a subsequent clinical trial that evaluated 46 pediatric patients with acute leukemia and reported that 3 or more repeated ZOL infusions offer a lower rate of transplant-related death, lower occurrence of relapses and absence of GvHD. Global disease-free survival is also improved (272).

The efficiency degree of donor γδ T cell *ex vivo* expansion is evidenced when the graft is depleted for αβ TCR, as this was able to induce a remarkable clinical recovery in 74 patients with acute and chronic leukemia, in which 43 achieved an objective response and 25 achieved complete remission, with no risk of recurrence and with improved survival after allo-HSCT (267). The subsequent follow-up of 153 patients with acute leukemia after allo-HSCT showed that γδ T cell-enriched graft, even inducing few complete remissions (36 patients), was able to confer a long-term survival advantage in patients who exhibited high γδ T cell frequency in the blood (268). Finally, ZOL + IL-2 *in vivo* stimulation after infusion of PBMC depleted for αβ T cells in 2 patients resulted in a higher *in vivo* expansion of donor γδ T cells and NK cells that induced complete remission in these patients (288).

It is important to highlight that many Phase I clinical trials are emerging to investigate γδ T cells as alternative axes in several established therapies since the available clinical and preclinical data suggest that γδ T cell-based strategies be combined with agents that better target these cells against the tumor. Therefore, several studies aiming at the optimization of γδ T cell antitumor reactivity through genetic engineering approaches are currently registered **(** **Table 3** **)**. The use of these lymphocytes as platforms for CAR (NCT02656147) and TEG (NTR6541) engineering can overcome many obstacles observed in conventional adoptive therapy with αβ T cells and NK cells, although they also have their limitations (215, 228). Finally, *in vivo* stimulation and *ex vivo* expansion are also being insistently evaluated in the context of allo-HSCT (NCT02508038, NCT03862833) and the γδ T cell product infusion (NCT03885076, NCT04008381, NCT04028440, NCT03533816) in the expectation that a safe, effective and tolerable method for the treatment of patients will be discovered.

**Table 3:** Currently registered γδ T cell-based clinical trials.

Disease or clinical condition	*N* intended inclusion	Interventions	Phase	Start	Status	Study identifier
***In vivo* stimulation (autologous)**						
ALL and AML	22	ZOL after haplo-HSCT depleted for αβ T cells/CD19^+^ B cells	I	January, 2016	Recruiting	NCT02508038
Eligible patients for HSCT	20	ZOL and IL-2	I	March, 2019	Recruiting	NCT03862833
***Ex vivo* expansion (autologous)**						
AML	20	PB collection and BM aspirate (OS)	NA	August, 2018	Recruiting	NCT03885076
Relapsed or refractory AML	38	γδ T cell infusion	I	September, 2019	Recruiting	NCT04008381
Relapsed or refractory CLL	6	γδ T cell infusion	I	October, 2019	Recruiting	NCT04028440
ALL, AML and CML	38	EAGD T cell infusion after HSCT	I	January, 2020	Recruiting	NCT03533816
**Genetic engineering**						
AML	18	TEG001	I	June, 2017	Recruiting	NTR6541
ALL and CLL	48	anti-CD19 CAR γδ T cells infusion	I	October, 2017	Not yet recruiting	NCT02656147

ALL, acute lymphoblastic leukemia; AML, acute myeloid leukemia; BM, bone marrow; CAR, chimeric antigen receptor; CLL, chronic lymphocytic leukemia; EAGD T cell, expanded/activated γδ T cells; haplo, haploidentical; HSCT, hematopoietic stem cell transplantation; IL, interleukin; NA, not applicable; OS, observational study; PB, peripheral blood; TEG, T cells engineered to express a defined γδ TCR; ZOL, zoledronate.

## Concluding Remarks and Outlooks for the Future

Through this review, we hope to shed light on a relatively unexplored unconventional T cell. Nonetheless, it is one that has proven to be an important component in the leukemic microenvironment, since it responds effectively against the tumor and is able to affect the clinical outcome in patients with leukemia, as we recently reviewed (289). γδ T cells have unique immunological properties that allow the development of an off-the-shelf immunotherapy with universal applicability, that is, independent of histocompatibility related factors since γδ T cells respond regardless of MHC/HLA expression and recognize Ags presented by ubiquitous monomorphic molecules in many tumors in humans.

Furthermore, the clinical responses reported in clinical and pre-clinical trials, already reviewed here, highlight the importance of further increasing γδ T cell reactivity, either by raising intracellular pAg concentrations to “sensitize” LCs or by projecting γδ T cells with higher expression of receptors associated with cytotoxicity, adhesion and homing, as this allows recirculation and immune surveillance in different tumor compartments, even under hypoxia. The fact that these cells predominate in the blood and healthy or malignant tissues provides a migratory advantage over αβ T cells or NK cells and a greater ability to infiltrate and respond in the leukemic microenvironment; in particular the Vδ1 subtype, which has improved cytotoxicity and resistance to exhaustion or AICD.

The difficulty that still needs to be overcome for the therapeutic use of these cells is, in fact, is that of how to obtain a clinically significant cell proportion. As such, new techniques for cell expansion (or improvement) are necessary. In addition, ensuring that γδ T cell antileukemic phenotype is not diverted by TME stimuli also represents another challenge to be faced. Therefore, the modulation and effective targeting of these cells need to be achieved. Finally, improving and maintaining their *in vivo* persistence and invigorating exhausted γδ T cells also represent additional barriers that can be reversed using molecular factors that support their cytotoxicity in TME *in vivo*. The fact is that the innate and adaptive γδ T cell properties will lead to advances in better antileukemic approaches and potentially establish which of these will provide a real and applicable translational perspective.

## Author Contributions

NDA and MSB established the initial conception, projected, and wrote this manuscript. NDA, MSB and TLPR collected, analyzed, and reviewed the data. MSB designed the illustrations and tables. NDA, FM-G, FSHA, AMT, AM and AGC supervised the project development, interpreted the data, and reviewed this manuscript. All authors contributed to the article and approved the submitted version.

## Funding

This work was funded by Fundação de Amparo à Pesquisa do Estado do Amazonas (FAPEAM) (Pró-Estado Program - #002/2008, PAPAC Program - #005/2019 and and POSGRAD Program-#006/2020), Conselho Nacional de Desenvolvimento Científico e Tecnológico (CNPq), Coordenação de Aperfeiçoamento de Pessoal de Nível Superior (CAPES) (PROCAD-Amazônia 2018 Program-#88881.200581/2018-01) and the Brazilian Ministry of Health. MSB, TLPR, NDA, FM-G and FSHA have fellowships from FAPEAM, CAPES and CNPq (SI and PhD students). AM is a level 2 research fellow from CNPq. The funders had no role in study design and decision to publish, or preparation of the manuscript.

## Conflict of Interest

The authors declare that the research was conducted in the absence of any commercial or financial relationships that could be construed as a potential conflict of interest.

## Publisher’s Note

All claims expressed in this article are solely those of the authors and do not necessarily represent those of their affiliated organizations, or those of the publisher, the editors and the reviewers. Any product that may be evaluated in this article, or claim that may be made by its manufacturer, is not guaranteed or endorsed by the publisher.
